# Exploring drivers of litter decomposition in a greening Arctic: results from a transplant experiment across a treeline

**DOI:** 10.1002/ecy.2442

**Published:** 2018-08-15

**Authors:** Thomas C. Parker, Jonathan Sanderman, Robert D. Holden, Gesche Blume‐Werry, Sofie Sjögersten, David Large, Miguel Castro‐Díaz, Lorna E. Street, Jens‐Arne Subke, Philip A. Wookey

**Affiliations:** ^1^ Biological and Environmental Sciences School of Natural Sciences University of Stirling Stirling FK9 4LA United Kingdom; ^2^ Department of Animal and Plant Sciences University of Sheffield Alfred Denny Building Sheffield S10 2TN United Kingdom; ^3^ Woods Hole Research Center 149 Woods Hole Road Falmouth Massachusetts 02540 USA; ^4^ Abisko Scientific Research Station Vetenskapens väg 38 Abisko SE‐981 07 Sweden; ^5^ Experimental Plant Ecology Institute of Botany and Landscape Ecology Greifswald University Greifswald 17487 Germany; ^6^ School of Biosciences University of Nottingham Sutton Bonington Campus Sutton Bonington LE12 5RD United Kingdom; ^7^ Department of Chemical and Environmental Engineering University of Nottingham Nottingham NG7 2RD United Kingdom; ^8^ School of GeoSciences University of Edinburgh Edinburgh EH9 3FF United Kingdom

**Keywords:** Arctic, decomposition, forest, litter, snow, tundra, vegetation change

## Abstract

Decomposition of plant litter is a key control over carbon (C) storage in the soil. The biochemistry of the litter being produced, the environment in which the decomposition is taking place, and the community composition and metabolism of the decomposer organisms exert a combined influence over decomposition rates. As deciduous shrubs and trees are expanding into tundra ecosystems as a result of regional climate warming, this change in vegetation represents a change in litter input to tundra soils and a change in the environment in which litter decomposes. To test the importance of litter biochemistry and environment in determining litter mass loss, we reciprocally transplanted litter between heath (*Empetrum nigrum*), shrub (*Betula nana*), and forest (*Betula pubescens*) at a sub‐Arctic treeline in Sweden. As expansion of shrubs and trees promotes deeper snow, we also used a snow fence experiment in a tundra heath environment to understand the importance of snow depth, relative to other factors, in the decomposition of litter. Our results show that *B. pubescens* and *B. nana* leaf litter decomposed at faster rates than *E. nigrum* litter across all environments, while all litter species decomposed at faster rates in the forest and shrub environments than in the tundra heath. The effect of increased snow on decomposition was minimal, leading us to conclude that microbial activity over summer in the productive forest and shrub vegetation is driving increased mass loss compared to the heath. Using *B. pubescens* and *E. nigrum* litter, we demonstrate that degradation of carbohydrate‐C is a significant driver of mass loss in the forest. This pathway was less prominent in the heath, which is consistent with observations that tundra soils typically have high concentrations of “labile” C. This experiment suggests that further expansion of shrubs and trees may stimulate the loss of undecomposed carbohydrate C in the tundra.

## Introduction

Climate warming in the Arctic of 1–4°C since 1960 (Serreze and Francis 2006, Serreze and Barry 2011) has resulted in large areas of tundra becoming more productive, with some landscapes showing increases in aboveground biomass of 10 g·m^−2^·yr^−1^ (Epstein et al. 2012). In many of these areas, shrubs and trees have been observed to increase in cover and height (Myers‐Smith et al. 2011, Elmendorf et al. 2012) and are generally thought to contribute to the increase in “greenness” that is observed from space (Tape et al. 2006). Earth system models have predicted that increased productivity in Arctic ecosystems will increase carbon (C) sequestration at the biome level (Cramer et al. 2001, Qian et al. 2010, Todd‐Brown et al. 2013) through increased litterfall. However, these predictions are at odds with observations in the Arctic of lower soil organic matter (SOM) storage under shrub and tree species than in adjacent tundra systems (Wilmking et al. 2006, Hartley et al. 2012, Parker et al. 2015). This suggests that we do not yet fully understand the interactions between plant functional types (PFTs), litter input, and decomposition rates and ecosystem carbon cycling in the Arctic.

Plant litter is the primary input of C into soil (Aber and Melillo 2001); its decomposition contributes toward humic substances that can lead to the formation of stable soil organic matter (SOM; Melillo et al. 1989, Sollins et al. 1996). Along with physicochemical environmental controls (i.e., temperature, humidity, pH, mineralogy), the species identity and functional type are key to determining the rate of decomposition of their litter and eventual contribution to SOM (Dorrepaal et al. 2005, Cornelissen et al. 2007, Cornwell et al. 2008, Brovkin et al. 2012). More specifically, the chemical composition of litter is important in determining its decomposition in any given environment (Coûteaux et al. 1995) with low carbon : nitrogen and high cellulose : lignin content favoring faster decomposition (Melillo et al. 1989). The decomposition of litter can be highly dependent on the interaction between litter species identity and the decomposer environment (Freschet et al. 2012, Keiser et al. 2014). Understanding the decomposition of different litter types in relevant contrasting environments will give insight into how litter decomposition may be altered under future global change.

*Empetrum nigrum* is widespread across Arctic and alpine tundras of Fennoscandia and boreal forests across Eurasia (Bell and Tallis 1973, Tybirk et al. 2000, Büntgen et al. 2014). Decomposition of *E. nigrum* leaf litter is very slow due to its production of allelopathic compounds (Wardle et al. 1998, Gallet et al. 1999) and high concentrations of the lipid polymer cutin, which is particularly slow to break down (Tegelaar et al. 1989, Rasse et al. 2005) as a result of a well‐developed waxy cuticle (Bliss 1962, Hetherington et al. 1984). In addition, its physical structure (small, needle‐like leaves with low specific leaf area (Tybirk et al. 2000, Kleyer et al. 2008, Kattge et al. 2011)), is also likely contribute to slow decomposition in the field. By contrast, leaf litter of deciduous shrubs and trees decomposes faster than that of evergreen species such as *E. nigrum* (Aerts et al. 2006, Cornwell et al. 2008, McLaren et al. 2017). Litter inputs are also known to stimulate the decomposition of SOM (Subke et al. 2004), in particular, high quality litter inputs from deciduous boreal systems are linked to faster biogeochemical cycling and lower soil carbon stocks than evergreen systems (Melvin et al. 2015). A replacement of ericaceous evergreen species with deciduous shrubs and forests could thus stimulate litter decomposition and eventually higher turnover of SOM.

Previous work at the arctic treeline has found that local site characteristics, specifically, the presence or absence of forest cover, exerted the strongest control on the decomposition of *B. pubescens* leaf litter, with higher rates of decomposition in birch forests than nearby tundra heaths (Sjögersten and Wookey 2004). This vegetation contrast was apparently more important than differences in regional climate (in contrast to the findings of other studies; Dorrepaal et al. 2005, Cornelissen et al. 2007) and experimental warming. The authors hypothesized that litter moisture in the birch forest was important in enhancing decomposition rates, but other abiotic factors such as deeper snow cover and therefore warmer winter soils and more active microbial communities (Grogan and Jonasson 2006, Blok et al. 2016) could also contribute to this. Contrasting decomposition rates between forest and tundra sites may therefore reflect the combined influence of several factors, both biotic and abiotic, the disentangling of which remains challenging.

Saprotrophic fungi that grow in litter horizons of forest floors have the capacity to degrade a large range of simple and complex plant‐derived structural molecules and are therefore key to the decomposition of litter (Hatakka 1994, Rytioja et al. 2014, Talbot et al. 2015). Decomposition in tundra soils, by contrast, may be under different controls, where strong environmental pressure, such as low temperature (Robinson 2001) and a “closed” C and N cycle dominated by ericoid mycorrhizal fungi (Read and Perez‐Moreno 2003), may restrict the growth and activity of other fungi. A comparison of the components of soil C in forest and tundra heath supports this view, showing that tundra has a more “labile” signature, with more poorly decomposed, cellulose‐related fractions than the soil of mountain birch forest (Sjögersten et al. 2003). This would suggest that there is less fungal activity in the tundra, especially that of “brown‐rot” fungi, which target cellulose as their primary energy source (Talbot et al. 2015). An expansion of forests could result in increased metabolism of previously poorly decomposed litter should the appropriate decomposer community become present.

Using a decomposition experiment whereby litter from the dominant species of three important vegetation types (forest, shrub, and tundra heath) was reciprocally transplanted across a sub‐Arctic treeline, we aimed to understand the key drivers of decomposition rates in this ecosystem. We tested the following specific hypotheses: (1) litter from the more productive vegetation types (forest and shrub) decomposes at the fastest rates, regardless of the local soil environment; (2) the forest and shrub environments are more favorable than tundra heath for the decomposition of all litter types, irrespective of origin; (3) deep winter snow and associated soil microclimates, which are characteristic of forest and shrub environments, increase litter decomposition compared to heath environments.

## Materials and Methods

### Sites description

The study area spans a 2‐km^2^, permafrost‐free landscape around the sub‐Arctic/alpine treeline at Nissunsnuohkki (Abisko area, Sweden; ~68°18′ N, 18°49′ E, ~600 m above sea level). The treeline is formed by mountain birch (*Betula pubescens* Ehrh. ssp *czerepanovii* (Orlova) Hämet Ahti), with an ericaceous understorey, and the ecotone typically comprises a thick layer of shrub vegetation before transitioning to tundra heath dominated by *Empetrum nigrum* L. ssp *hermaphroditum* (Hagerup) Böcher and *Vaccinium vitis‐idaea* L. The intermediate shrub zone is dominated by *Betula nana* L. and grey willow (*Salix*) species (typically *Salix glauca*, often accompanied by *Salix lanata*; other *Salix* spp., including *S. hastata* and *S. lapponum*, occur less frequently). Soil pH in the organic horizon is 4.5 ± 0.1 at forest and 4.3 ± 0.1 at heath locations in the Abisko area (mean ± SE; Table 1). Twelve independent, short (<100 m) transects were established across the multiple forest patches in the treeline study area. Transect lengths ranged from 52 to 97 m depending on the sharpness of the forest–heath ecotone transition. The soils at all sites are well drained (Sjögersten and Wookey 2002) with standing water only observable for a short number of days every year at snow melt (T. C. Parker, *personal observation*). Care was taken to select vegetation transitions that were not influenced by local topography, for example where water and snow accumulation due to dips and hollows dominate site conditions, and avoiding steep slopes (mean elevation change from heath to forest plots of 2.7 m). For more details on study sites, see Parker et al. (2015).

**Table 1:** Site characteristics along transects at Abisko, Sweden

Property	Heath	Shrub	Forest
Vegetation			
Distance from heath (m)		28.3 ± 2.9	67.6 ± 5.9
Canopy height (cm)	14.7 ± 0.7	32.0 ± 2.4	19.0 ± 1.7
*Betula pubescens* density (trees/ha)			785.0 ± 109.0
*Betula nana* cover (%)	21.2 ± 2.7	60.3 ± 4.8	8.0 ± 2.2
*Empetrum nigrum* cover (%)	65.4 ± 3.3	66.9 ± 4.7	45.4 ± 4.2
Soil			
pH (organic horizon)	4.3 ± 0.1	4.4 ± 0.1	4.5 ± 0.1
Organic horizon carbon (kg/m^2^)	7.0 ± 0.8	3.0 ± 0.5	2.0 ± 0.3
Mineral horizon carbon (kg/m^2^)	2.0 ± 0.3	3.3 ± 1.3	2.5 ± 0.4
2012–2013			
Summer temperature (°C)	5.4 ± 0.3	5.1 ± 0.3	5.5 ± 0.2
Winter temperature (°C)	−3.9 ± 0.2	−1.3 ± 0.2	−1.1 ± 0.2
2013–2014			
Summer temperature (°C)	6.6 ± 0.3	6.6 ± 0.6	7.1 ± 0.2
Winter temperature (°C)	−2.5 ± 0.5	−1.0 ± 0.1	−0.2 ± 0.1
Snow			
2012–2013			
Snow depth at transects	13.1 ± 1.8	35.4 ± 4.0	46.8 ± 3.4
Snow depth at snow fences (cm)	13.9 ± 2.2	22.6 ± 2.9	58.5 ± 13.3
2013–2014			
Snow depth at transects	14.4 ± 3.5	29.7 ± 5.3	72.2 ± 9.1
Snow depth at snow fences (cm)	13.0 ± 1.5	39.0 ± 8.7	78.2 ± 10.4

*Notes*

Values are means ± SE, *n* = 12. “Canopy height” refers to the actual vegetation canopy for heath and shrub communities and the understorey of the forest (where mountain birch trees, *Betula pubescens*, comprise the canopy). Snow depths measured over transects are paired in either 2013 or 2014 with snow depth data from the snow fence experiment, at plots that were selected to mimic snow depth along the transect. Vegetation and soil data (except temperature data) are adapted from Parker et al. (2015). Soil temperature data are average seasonal temperatures at 5 cm depth across 6 of the 12 transects. The start of each season is defined by soil temperatures deviating and remaining above (summer) or below (winter) 0°C.

Three plots (approximately 2 m^2^) were established along each transect in order to represent the transition in vegetation from heath to forest. These were designated: tundra heath (H), shrub (S), and forest (F; see Table 1 for further plot details). H plots were chosen for an open heath environment with low *B*. *nana* cover and a low canopy height and with vegetation dominated by *E. nigrum*. S plots were identified as areas dominated by *B. nana* with shrub height characteristically between 40 and 60 cm. F plots were chosen to be in areas dominated by *B. pubescens*, approximately 10 to 15 m inside the forest edge.

### Snow fences and snow depth measurements

Five replicate, 3.5 m wide, 1.5 m high, snow fences were erected on tundra heath sites between 0.1 and 1 km north of the transect sites (Appendix S1: Fig. S1). They were erected before snowfall in 2012 and in 2013 (and lowered during the summer to avoid shading the vegetation and influencing evapotranspiration), and designed to create snow drifts of comparable depth to the typical seasonal snow cover at F and S plots on the transects. To replicate the snow at F plots, plots were set up 2 m to the leeward side of the fence, 7 m for the S plots, and 20 m for the H plots (no extra snow). Snow depths were measured at both snow fence and transect plots, once each between 14 March and 29 March in 2013 and between 29 March and 30 March in 2014. At each of the transects, snow depth was recorded at five points taken within 1.5 m of the logged position of the litter bags (the horizontal accuracy of the GPS unit was 3 m). At the snow fences this was not necessary due to the exact known location of the litter bags under the snow, and one measurement was taken per plot. The snow fence treatment that replicated shrub snow depths increased snow depths by 17 cm (compared to 19 cm in the shrub sites). The snow fence plots that replicated snow found in the forests increased snow depth by 55 cm (compared to 46 cm in the forest sites; Table 1).

### Litter bags

Litter was collected from four different transects at the Abisko study site from 2 September 2012 to 12 September 2012. Freshly fallen *B. pubescens* and *B. nana* litter was collected from the top of the litter layer, taking care to exclude older litter (which was easily identified). *E. nigrum* litter was collected by carefully removing senesced leaves from the stem of extracted *Empetrum* shoots. Only recently senesced leaves were taken (light brown color, 2–4 yr old according to growth scars). Litter was collected from the “home” plots in which each species is dominant; i.e., *B. pubescens* from F plots, *B. nana* from S plots, and *E. nigrum* from H plots. All litter was sorted to remove any adhering particles or litter from other species, and air dried at 40°C for 72 h. For each species, 0.5 ± 0.01 g of litter (mean ± SE) was weighed into 7 × 7 cm polyester mesh bags with a 0.3 mm mesh size and heat sealed. Note that the relatively small mesh size required to contain the *E. nigrum* litter will exclude many soil and litter fauna. All litter bags were placed in the field on 17 September 2012. Six bags of each species were placed at every plot on all 12 transects and at snow fences. Care was taken to ensure that every bag had good contact with the L horizon at each plot. Two corners of each bag were fastened to the ground using stainless steel pins and all bags were tied with nylon thread to nearby vegetation. Bags were also deployed in the same manner on the leeward side of the snow fences. Ten additional 0.5‐g samples of each species were oven dried at 60°C for 72 h, and the mass of undecomposed litter at the initiation of field emplacement was corrected according to the residual moisture of air‐dried litter.

On 13 June 2013 (269 d of incubation), 24 July 2013 (310 d), 16 September 2013 (365 d), 20 June 2014 (641 d), and 18 October 2015 (1,126 d), one litter bag of each species (one to two on the final harvest, see later in the current section) at each plot at both transect and snow fence sites was retrieved from the field and oven dried at 60°C for 72 h. Once ingrown vegetation was removed, the remaining litter was extracted, weighed, and the percentage of mass remaining was calculated. Due to the duration of field emplacement (>3 yr) some litter bags were lost or disturbed (9.8%); at the final harvest, if two bags were remaining at a plot and both bags were not damaged, a mean percentage remaining of the two was calculated.

### Solid state CPMAS ^13^C NMR

Five samples of *B. pubescens* and *E. nigrum* in either the H or F sites at the 641‐d harvest were taken forward for solid state ^13^C nuclear magnetic resonance CPMAS ^13^C NMR (cross‐polarization/magic angle spinning ^13^C nuclear magnetic resonance spectroscopy) and elemental (C and N) analysis. Samples were randomly selected from a pool of 12 samples within each of the four groups (species [*B. pubescens*,* E. nigrum*] and site [forest, heath] combinations). For both species, three randomly selected undecomposed litter samples (from a pool of 10 undecomposed samples at the beginning of the experiment) were taken forward for CPMAS ^13^C NMR. This totaled 26 samples taken for CPMAS ^13^C NMR. *Betula pubescens* and *E. nigrum* were selected for the for CPMAS ^13^C NMR analysis as they had the most contrasting decomposition rates.

CPMAS ^13^C NMR spectra were obtained using a Bruker Avance 300 spectrometer (Bruker Analytik GmbH, Rheinstetten, Germany). A total of 2500 scans were obtained from approximately 0.25 g of ball‐milled leaf material of each sample, packed into a cylindrical zirconia rotor with approximately 0.02 g Tetrakis (trimethylsilyl) silane (TKS) packed on top and sealed with a Bruker Kel‐F drive cap (Bruker Analytik GmbH). The scanning parameters were as follows: 200 MHz frequency, 1,000 ms contact time, 1.5 s relaxation time, 5,500 Hz spinning speed, and line broadening of 50 Hz. Chemical shift values were obtained compared to TKS. Total signal intensities from NMR spectra were integrated into eight major chemical shift regions (Table 3).

### FTIR‐NMR spectra transformation

Diffuse reflectance Fourier transform infrared (FTIR) spectroscopy in combination with multivariate statistical techniques represents a robust and low‐cost way of predicting major properties of various materials including NMR‐observed chemistry (Forouzangohar et al. 2015). We applied FTIR spectroscopy to build a predictive model from the 26 samples with NMR spectra. This model was later used to predict change in litter organic chemistry for the final harvest. For these 26 samples, FTIR spectra were acquired on a Bruker Vertex 70 (Bruker Optics, Billerica, Massachusetts, USA) equipped with a wide‐range Si beam splitter and mid infrared detector with Csl windows and a Pike Autodiff (Pike Technologies, Madison, Wisconsin, USA) diffuse reflectance accessory for finely ground samples from undecomposed and 641‐d harvests, which already had associated NMR spectra (*n* = 26), as well as on 20 samples from the 1,126‐d harvest that did not have associated NMR spectra. Consistent with the sample selection for NMR, five replicates of each treatment were randomly selected from the 1,126‐d harvest (*n* = 20). Spectra were acquired on finely ground material over 6,000–180 per cm with a resolution of 4 per cm. For each sample, 60 scans were collected and averaged using the OPUS software package (Bruker Optics) and then corrected for background signal (average of 60 scans) and transformed into absorbance spectra.

The acquired FTIR spectra were truncated to 4,000–630 per cm and normalized using the standard normal variation (SNV) transformation. A partial least squares regression (PLSR) analysis was used to predict the eight major NMR chemical shift regions on the 26 samples that had associated NMR data. Given the small sample size (*n* = 26), a full cross‐validation procedure was used. The PLSR analysis was able to produce good five‐factor models for the dominant chemical shift regions, with less reliability for the regions with only minor contributions (Appendix S1: Table S1). These models were then used to predict the signal intensity in each chemical shift region, along with prediction errors (De Vries and Ter Braak, 1995), for the unknown samples that decomposed for 1,126 d in the field. All data processing and analysis was performed using the Unscrambler X software (CAMO Software AS, Oslo, Norway). To aid in the interpretation of the ^13^C NMR data, the distribution of signal intensity from each of the chemical shift regions (Table 3) at each time point (undecomposed, 641 d, and 1,126 cd) was used in a molecular mixing model (Baldock et al. 2004) that calculates the best linear fit of the distribution of NMR signal intensity of five major biochemical components (carbohydrates, protein, lignin, lipids, and carboxyl C).

After analysis by CPMAS ^13^C NMR (undecomposed and 641 d), samples were separated from TKS, ensuring no contamination of the sample, and were analysed for carbon and nitrogen content after combustion in a Vario EL Cube elemental analyzer (Elementar, Hanau, Germany). After FTIR analysis, the 1,126‐d samples were analysed for carbon and nitrogen content using a Flash 2000 CN analyzer (Thermo Scientific, Waltham, Massachusetts, USA). The carbon content data were then applied to the actual mass of the litter remaining and estimated fractions of C components to calculate the mass of carbon remaining in each component.

### Statistical analysis

Decay constants (*k*) were calculated for the loss of litter mass of every replicate species and site combination on both the snow fence and natural transect experiments according to the negative exponential litter decay model

(1) $$\ln(M_{t}/M_{0})=-kt$$

where *M*_0_ is the initial dry mass of the sample and *M*_*t*_ is the mass at time *t* (yr). The first two harvests (269 and 310 d) were omitted for this calculation because they do not fit the long‐term exponential decay model as a result of low mass loss in the first winter. Differences in *k* between site (heath, shrub, and forest (or snow level in the case of the snow fence experiment) and species (*E. nigrum*,* B. nana*, and *B. pubescens*) were compared using a linear mixed effects model in the nlme package (Pinheiro et al. 2017) of the R statistical software (R Development Core Team 2016). In the linear mixed effects model, transect was expressed as a random intercept factor due to unquantified baseline differences in decomposition between transects. The interaction between site and species was found not to be statistically significant in the original model (*P *=* *0.64) and was therefore removed from the analysis (Crawley 2007). Pairwise comparisons of decomposition rates between different levels of species and site types were carried out by comparing Least‐Square means derived from the statistical models with a Tukey HSD test.

The mass remaining and the percentage of undecomposed samples remaining of carbohydrates, lipids, and lignin estimated from NMR spectra were analysed using a three‐way ANOVA with time, site (heath and forest), and species (*B. pubescens* and *E. nigrum*) as treatment effects. The percentage data were arcsine‐square‐root transformed prior to analysis. All analyses were carried out using R v3.3.1. (R Development Core Team 2016).

## Results

### Litter decomposition rate

**Figure 1:**
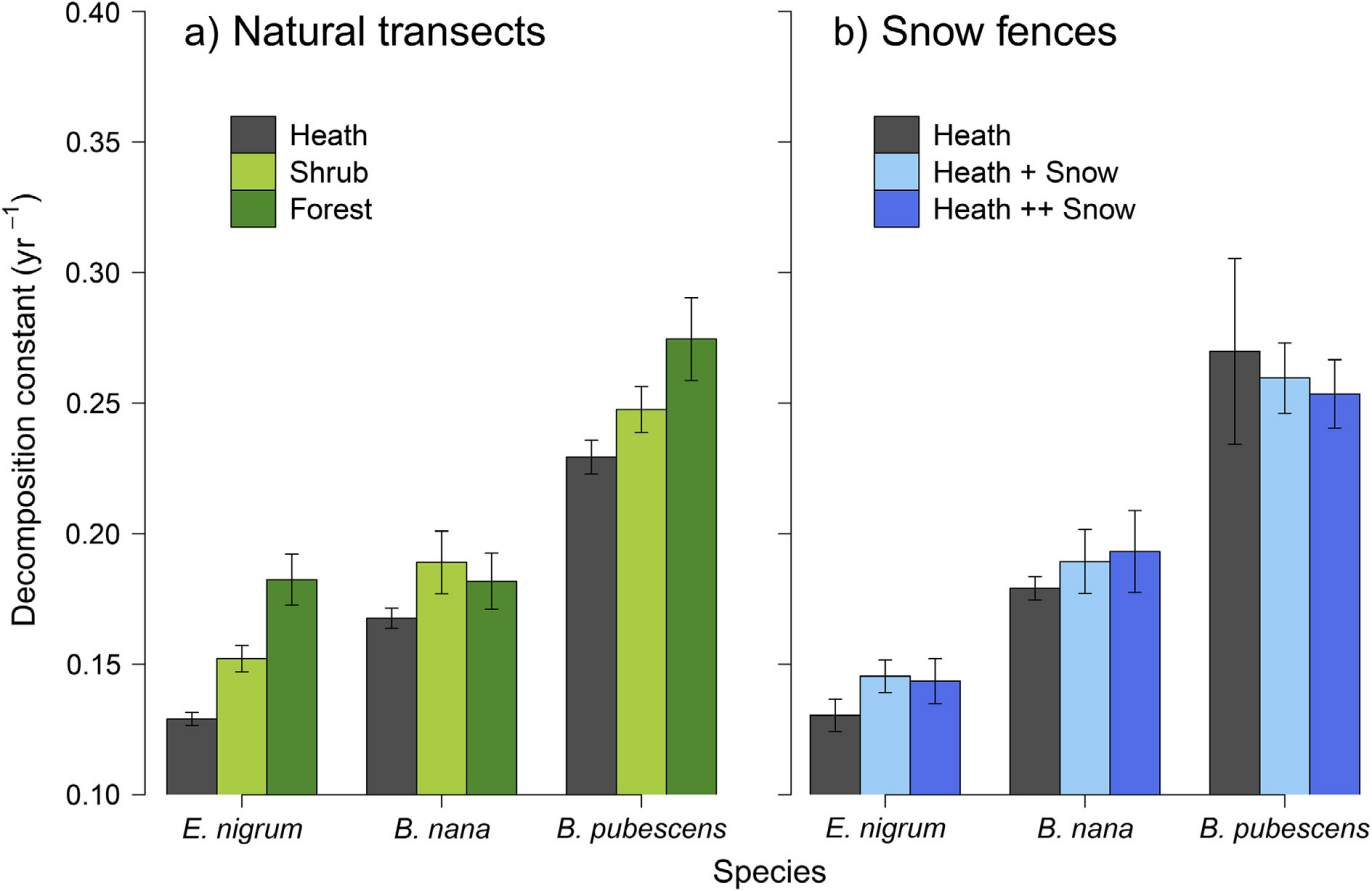
Decomposition constants (*k*) of *Empetrum nigrum*,* Betula nana*, and *Betula pubescens* litter across (a) transects across natural treelines from heath to forest and (b) under three different snow depths simulating snow accumulation found at different vegetation types: Heath (control), + Snow (Shrub) and ++ Snow (Forest). Error bars represent ± SE (transects *n* = 12, snow fences *n* = 5).

**Figure 2:**
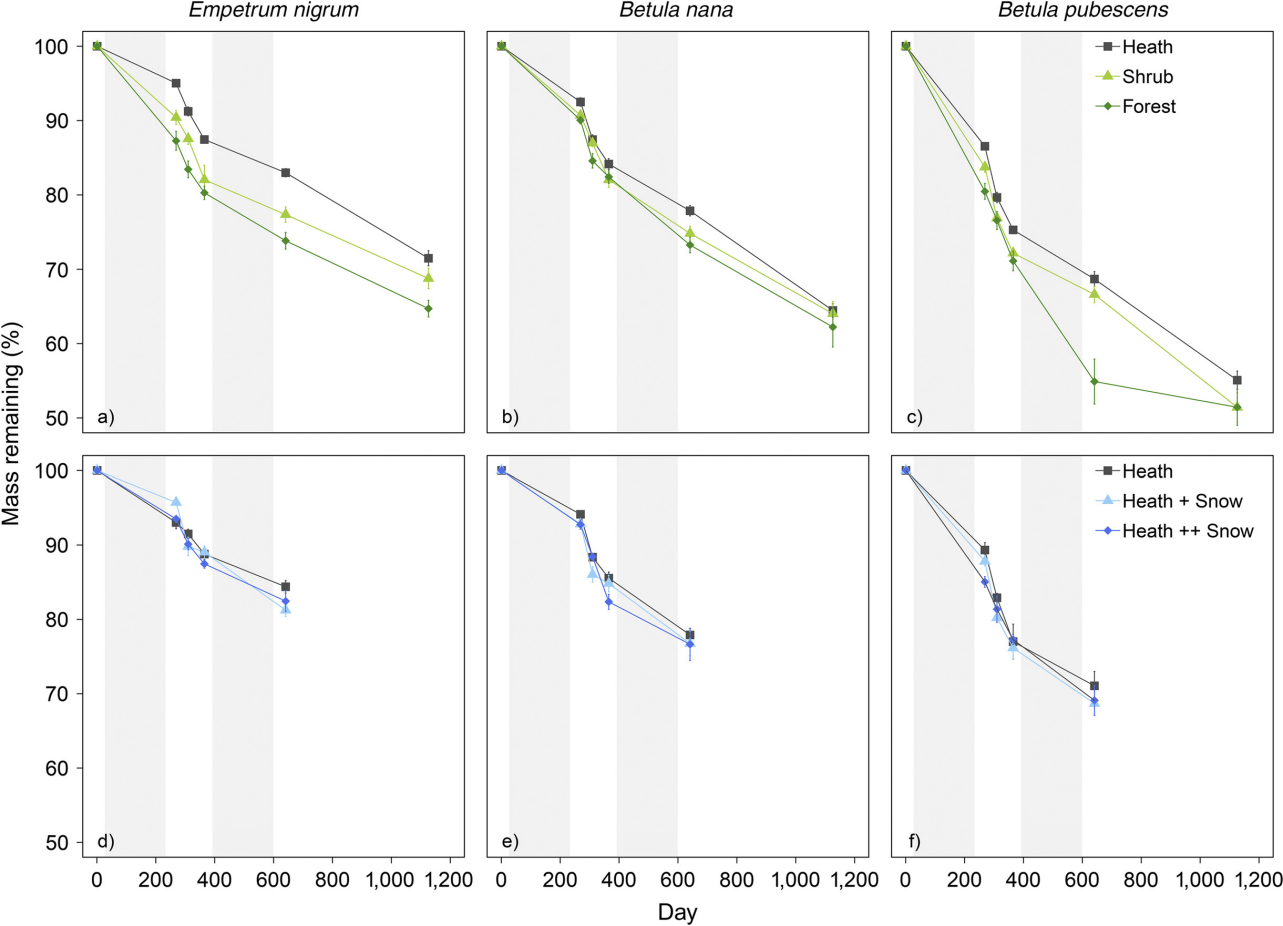
Percentage of litter mass remaining over time for three different species: (a, d) *Empetrum nigrum*, (b, e) *Betula nana*, (c, f) *Betula pubescens* in either distinct vegetation communities (heath, shrub, or forest), distributed across natural transects (a, b, c), or under three different snow depths simulating snow accumulation found at different vegetation types: Heath (control), + Snow (Shrub) and ++ Snow (Forest) (d, e, f). Error bars represent ± SE (transects *n* = 12, snow fences *n* = 5). The extent of the shaded areas on the *x*‐axis indicates the length of the snow covered season in the first two years of the study.

Decomposition rates differed significantly between species on both the natural transects (*P *<* *0.001, Table 2) and at the snow fence experiment (*P *<* *0.001, Table 2). *Betula pubescens*, with an average decomposition constant of 0.25 per yr across all sites, decomposed significantly faster than both *B. nana* (0.18 per yr; *P *<* *0.001) and *E. nigrum* (0.15 per yr, *P *<* *0.001, Fig. 1a), *B. nana* decomposed faster than *E. nigrum* (*P *=* *0.0018). The host site (in which litter was decomposing) was also highly significantly related to decomposition rates in the litter transplant experiment (*P *<* *0.001, Fig. 1a, Table 2). On average, across litter types, litter decomposed marginally faster in the forest (decomposition constant = 0.21 per yr) than in the shrub sites (0.20 per yr, *P *=* *0.06) and heath sites (0.18 per yr, *P *<* *0.001). Overall, decomposition was faster in the shrub sites than the heath sites (*P *=* *0.011). There was no effect of different snow treatments on litter decomposition rates in the snow fence experiment (*P *=* *0.9, Fig. 1b, Table 2). At the end of the experiment, *B. pubescens* in the forest and shrub plots had the least mass remaining (51% each; Fig. 2) and *E. nigrum* in the heath had the most (71%; Fig. 2).

**Table 2:** The effect of species of litter and incubation site on decomposition rate (*k*) on the natural transects (“Site” represents differences both in abiotic factors (e.g., snow cover, thermal and moisture regimes) and biotic factors (e.g., microbial community and others) and at the snow fences (where “Environment” initially represents differences in abiotic factors associated with altered snow only)

Factor	df	*F*	*P*
Natural transects			
Species	2,89	94.4	<0.001
Site	2,89	13.3	<0.001
Snow fence experiment			
Species	2,36	86.9	<0.001
Snow	2,36	0.2	0.9

### ^13^C NMR and carbon components of litter

Prior to decomposition, *E. nigrum* and *B. pubescens* differed substantially in the relative contributions of different regions of their NMR spectra, with *E. nigrum* dominated by alkyl‐containing compounds and *B. pubescens* dominated by O‐alkyl‐containing compounds (Table 3). These initial proportional differences in NMR spectra were still apparent after litter had decomposed after 641 and 1,126 d in the field (Table 3). The proportion of O‐alkyl compounds in both litter types reduced through time while alkyls remained stable as a proportion of the litter remaining in both litter types, resulting in an increase in alkyl : O‐alkyl ratio (Table 3). The C:N ratio of fresh *B. pubescens* litter was (60.8) under one‐half of that measured in *E. nigrum* (138.3). Over time, the C:N ratio decreased rapidly for both litter types, especially in the forest plots where C:N ratio at the end of the experiment reduced to 23.6 and 50.8 in for *B. pubescens* and *E. nigrum* respectively (compared to 31.9 and 64.3 at the heath plots; Table 3).

**Table 3:** Percentage contributions of chemical shift regions to ^13^C NMR spectra, Alkyl : O‐Alkyl ratios, and C:N ratios of litter samples of *Betula pubescens* and *Empetrum nigrum* that were decomposing in forest or heath environments at 0 d (undecomposed), 614 d, and 1,126 d

Parameter	0 d	641 d		1,126 d	
*Betula pubescens*					
Alkyl (0–45 ppm)	15.5 ± 0.3	20.9 ± 1.2	18.5 ± 0.2	20.8 ± 1.4	25.8 ± 7.3
N‐Alkyl/Methoxyl (45–60 ppm)	5.1 ± 0.1	6.6 ± 0.6	6.2 ± 0.1	6.6 ± 0.1	6.6 ± 0.2
O‐Alkyl (60–95 ppm)	47.6 ± 0.9	38.3 ± 1.6	45.7 ± 0.7	38.3 ± 1.0	40.2 ± 4.8
Di‐O‐Alkyl (95–110 ppm)	11.3 ± 0.2	8.7 ± 0.5	10.4 ± 0.1	8.7 ± 0.3	8.9 ± 1.2
Aryl (110–145 ppm)	11.1 ± 0.8	11.4 ± 0.8	9.6 ± 0.3	11.3 ± 0.4	9.4 ± 0.6
O‐Aryl (145–165 ppm)	4.2 ± 0.2	4.5 ± 0.8	3.6 ± 0.3	4.7 ± 0.4	3.1 ± 0.5
Amide/Carboxyl (165–190 ppm)	5.1 ± 0.3	9.5 ± 1.9	6.0 ± 0.4	9.7 ± 0.7	5.9 ± 1.1
Alkyl/O‐Alkyl	0.3 ± 0.0	0.5 ± 0.0	0.4 ± 0.0	0.5 ± 0.0	0.8 ± 0.4
C:N	60.8 ± 4.3	31.5 ± 1.9	49.7 ± 0.9	23.6 ± 1.3	31.9 ± 3.2
*Empetrum nigrum*					
Alkyl (0–45 ppm)	43.9 ± 1.0	50.3 ± 1.7	51.6 ± 1.3	52.3 ± 2.3	54.4 ± 0.9
N‐Alkyl/Methoxyl (45–60 ppm)	4.7 ± 0.2	5.0 ± 0.3	5.5 ± 0.2	6.0 ± 0.1	6.0 ± 0.1
O‐Alkyl (60–95 ppm)	26.9 ± 1.0	21.4 ± 1.1	24.8 ± 0.6	21.3 ± 1.7	21.7 ± 0.5
Di‐O‐Alkyl (95–110 ppm)	6.2 ± 0.1	4.8 ± 0.4	5.0 ± 0.1	4.4 ± 0.5	4.4 ± 0.2
Aryl (110–145 ppm)	9.9 ± 0.1	9.6 ± 0.3	7.9 ± 0.4	8.7 ± 0.2	7.9 ± 0.1
O‐Aryl (145–165 ppm)	3.9 ± 0.3	4.0 ± 0.4	2.4 ± 0.3	2.9 ± 0.1	2.4 ± 0.1
Amide/Carboxyl (165–190 ppm)	4.4 ± 0.3	4.9 ± 0.5	2.8 ± 0.6	4.3 ± 0.1	3.2 ± 0.2
Alkyl/O‐Alkyl	1.6 ± 0.1	2.4 ± 0.2	2.1 ± 0.1	2.6 ± 0.4	2.5 ± 0.1
C:N	138.3 ± 3.0	74.6 ± 4.5	111.6 ± 5.0	50.8 ± 3.9	64.3 ± 3.1

*Notes*

Values are means ± SE (*n* = 5 for decomposed field samples, *n* = 3 for undecomposed samples).

**Figure 3:**
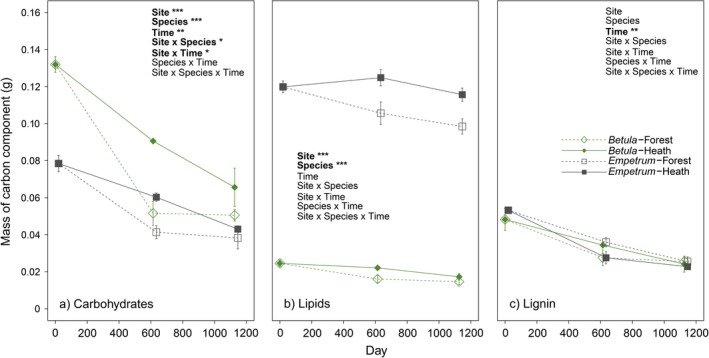
Mass of (a) carbohydrates, (b) lipids, and (c) lignin in *Betula pubescens* (green diamonds) and *Empetrum nigrum* litter (gray squares) in forest (open shapes) and heath (closed shapes) environments at initial levels (0 d), and after 614 and 1,126 d of decomposition (*t*5). Error bars represent ± SE (initial litter, *n* = 3; decomposed samples, *n* = 5). Boldface lettering in the inset text indicates significant (*P *<* *0.05) factors and interactions in three‐way analysis of variance; number of asterisks indicate level of significance: ***P <* *0.001, **P <* *0.01, *P <* *0.05. See Appendix S1: Table S2 for further statistics relating to these data.

Prior to decomposition, litter from *B. pubescens* contained 1.7 times more carbohydrate C than *E. nigrum*, whereas *E. nigrum* had 4.9 times more lipid C in its biomass compared to *B. pubescens*. Amounts of lignin were similar between the litter types (Fig. 3). After incubation in the field, there was a highly significant effect of site (*F* = 28, df = 1,32, *P *<* *0.001; Appendix S1: Table S2) and species of litter (*F* = 26, df = 1,32, *P *<* *0.001; Appendix S1: Table S2) on the mass of carbohydrates remaining in litter, whereby this mass was lower in litter decomposing in forest plots and *B. pubescens* contained higher amounts of carbohydrates than *E. nigrum*, respectively (Fig. 3a). In the forest, litter carbohydrates initially decomposed rapidly between 0 and 614 d, and then stabilized at approximately 40% (*B. pubescens*, Appendix S1: Fig. S2a) and 50% (*E. nigrum*, Appendix S1: Fig. S2a), after which there was only marginal mass loss (Fig. 3). In contrast, the decomposition of litter carbohydrates in the heath followed a more linear pattern, with slower decomposition to 614 d, which then continued to 1,126 d. The final percentage mass remaining of carbohydrates of both *B. pubescens* (49 %) and *E. nigrum* (54%) at the end of the experiment in the heath was within 10% and 6%, respectively, of the litter in the forest, despite slower initial decomposition rates (Appendix S1: Fig. S2a).

Due to very high alkyl‐C contents in *E. nigrum* litter, the mass of lipids modelled to be present in this litter was also very high (Fig. 3b), resulting in a highly significant relationship between species type and mass of lipids in extracted litter samples (*F* = 690, df = 1,32, *P *<* *0.001). There was also a strong effect of site on mass of lipids, with lower amounts remaining in both *E. nigrum* and *B. pubescens* at the forest plots (*F* = 15, df = 1,32, *P *<* *0.001; Appendix S1: Table S2). When expressed as a proportion of the original lipid mass, the results show a strong effect of species (*F* = 18, df = 1,32, *P *<* *0.001; Appendix S1: Table S2) and site (*F* = 12, df = 1,32, *P *=* *0.002; Appendix S1: Table S2); *B. pubescens* had 60% of lipid mass remaining in the forest and 70% in the heath, whereas *E. nigrum* had 82% remaining in the forest and 96% in the heath (Appendix S1: Fig. S2b).

Lignin was present in low amounts in litter (Fig. 3c) and there were no significant differences in mass of lignin remaining over the study duration between site (*F* = 0.4, df = 1,32, *P *=* *0.5; Appendix S1: Table S2) or species (*F* = 0.0003, df = 1,32, *P *=* *0.98; Appendix S1: Table S2), but there was a significant decline in mass with time (*F* = 11, df = 1,32, *P *=* *0.002; Appendix S1: Table S2). Although initial amounts of lignin were low (Fig. 3c), it decomposed in all species–site treatments to about 50% of its original amount (Appendix S1: Fig. S2c).

## Discussion

The greater decomposition rates of *B. pubescens* and *B. nana* than *E. nigrum* regardless of decomposition environment clearly support the first hypothesis that litter from an Arctic tree and shrub species decomposes at a faster rate than the typical heath species, *E. nigrum*. This difference is consistent with the differences in C stocks in the environments that these species dominate, respectively, i.e., low C stocks in forest and high C stocks in tundra heath (Hartley et al. 2012, Parker et al. 2015).

Litter of *E. nigrum*, a key species of tundra heaths, decomposed very slowly. This is likely due to high levels of aliphatic compounds (alkyls), which make up the lipids of its waxy cuticle (Bliss 1962, Hetherington et al. 1984). Lipid levels in *E. nigrum* litter were over four times higher than in *B. pubescens*, and showed very low rates of mass loss, especially in the tundra heath environment. While our methods cannot distinguish between plant‐ vs. microbe‐derived alkyls (Baldock et al. 1997), it is clear that these compounds are contributing substantially to the persistence of *E. nigrum* litter in this experiment. The strong contribution of lipids to long‐term SOC storage in tundra heaths is also corroborated by the components of C found in the SOM of ericaceous tundra around Abisko (Sjögersten et al. 2003), which also contained high levels of alkyls. This link between aliphatic compounds in *E. nigrum* litter and a resulting alkyl signature in the soil has also been found in Norwegian tundra heath systems (Väisänen et al. 2015), emphasizing that this could be a significant driver of high SOM storage in tundra. Although we could not explicitly address the potential role of the physical structure of the litter studied here, it is important in determining decomposition rates (Cornelissen et al. 1999). *E. nigrum* has a far lower surface area: mass ratio (specific leaf area) than the *Betula* species used in this study (Kleyer et al. 2008), which may render the substrate more immediately available to decomposer communities.

In contrast to *E. nigrum*,* B. pubescens* lost substantial mass in the initial stages of decomposition. The measurements of remaining carbon suggest that this initial rapid decomposition was due to the metabolism and breakdown of the initially high levels of carbohydrates (predominately O‐alkyls). This loss of carbohydrates is a likely contributing factor to rapid turnover of C and ultimately low storage of C in the soil in deciduous Arctic and boreal ecosystems (Melvin et al. 2015, Parker et al. 2015). Carbohydrates in *B. pubescens* litter decomposed to a similarly low residual level in the tundra as in the forest, even though their initial decomposition was not as rapid. This supports the hypothesis that litter identity is central to its eventual decomposability (Coûteaux et al. 1995, Cornelissen et al. 2007), irrespective of in situ processing rates.

We also examined the decomposition rates of leaf litter from *B. nana*, a shrub species that has been observed to be expanding its range over arctic tundra in response to climate change (Tape et al. 2006, Myers‐Smith et al. 2011). This litter also lost significantly more mass than *E. nigrum*, and observations of high soil C flux from these shrub systems (Parker et al. 2015) may in part be explained by this more rapid leaf litter turnover. However, *B. nana* decomposed at slower rates than *B. pubescens*, which could be due to a number of factors including differences in specific leaf area (a facet of physical structure; note earlier paragraph), N content, and structural C compounds. Indeed, with regard to the litter chemistry Väisänen et al. (2015) reported carbohydrate concentration of 39% and alkyl to O‐alkyl ratio around 0.51 indicating that the intermediate decomposition rates of *B. nana* may be attributed to its intermediate levels of carbohydrates (Väisänen et al. 2015). Based on our observed species‐specific decomposition rates, any expansion of *B. pubescens* forests is likely to increase leaf litter decomposition in tundra to a greater extent than an expansion of *B. nana*, but both are likely to increase C cycling rates if only PFT (deciduous) of the litter input is considered.

The second overarching hypothesis of this study, that litter would decompose fastest in the forest and shrub environments compared with the heath, was supported by the majority of the data, with the exception of the shrub *B. nana*. Our snow fence experiment gives some insight into separating the influence of abiotic (snow depth, temperature and, potentially, moisture) effects on decomposition from the confounding biological factors (i.e., vegetation/microbial). There were no increases in litter loss with increased winter snow depth over the 2 yr of study, concurring with findings of another study in arctic tundra (DeMarco et al. 2014) but not those of Blok et al. (2016). As the experimentally manipulated snow depth did not influence decomposition rates, we must conclude that the naturally deep snow cover was not the driver behind the rapid decomposition that we observed in the forest. We however, do not rule out a longer‐term effect of many years of snow cover on microbial communities and resulting decomposition rates. Litter moisture is an abiotic factor that we could not take directly into account in the present study. It is known to be important in controlling microbial activity and litter turnover in boreal forests (Schimel et al. 1999), and low surface moisture in heath ecosystems has been implicated in slowing decomposition (Sjögersten and Wookey 2004). We acknowledge that there are abiotic controls other than snow depth that we have not accounted for, but conclude that the major differences in decomposition that we observe along the treeline are due to microbial and biochemical differences.

We propose that the rapid decomposition of carbohydrate rich litter in the forest was driven by two interlinked processes: First, there is a rich and active fungal community (especially brown‐rot fungi) in the litter horizons of the forest (Lindahl et al. 2007) capable of producing an array of enzymes that can target initially available cellulose‐related structures (Talbot et al. 2015) until this source of C is depleted. Secondly, there is a biochemically favorable environment that “primes” the decomposition of cellulose in the forest plots due, in part, to the high‐cellulose content of previous litter falls. Temperature (Pietikainen et al. 2005) and pH (Rousk and Bååth 2011) are important in determining fungal and bacterial growth rates, but soil pH and thaw‐season soil temperature is remarkably similar across the study ecotones (Table 1). This leaves the biochemical environment as a key remaining factor explaining why fungi may grow well in the birch forests. Experimental additions of cellulose have been found to increase fungal growth (Subke et al. 2004, Meidute et al. 2008) and enzyme production (Talbot and Treseder 2012). Thus, it is feasible that, in the mountain birch forests in the present study, there are tight linkages between the carbohydrate‐rich litter, increased fungal activity, and rapid turnover of C (Parker et al. 2015).

The production of allelopathic compounds by *E. nigrum* is a process that can have ecosystem‐wide influence (Wardle et al. 1998). Production of poly‐phenolic secondary compounds by *E. nigrum* has been linked to inhibited activity of soil fungi and animals and, as a result, lowered decomposition rates and increased build‐up of SOM (Wardle et al. 1998, Tybirk et al. 2000). Slow decomposition rates of *E. nigrum* in the present study may partially be due to remaining residues of allelopathic compounds on the litter and in the surrounding litter in the heath. However, it should be noted that the forest sites also have high cover of *E. nigrum* across the understory (Parker et al. 2015) yet carbon turnover is very high compared with the heath. Although assessing the importance of allelopathy across the sub‐Arctic treeline is not in the scope of this work, it may have important controls over decomposition.

*Betula pubescens* litter in the forest plots decomposed to one‐half of its original mass within 18 months, with limited further mass loss for the remainder of the time in the field. This is consistent with observations that the most labile components of litter are decomposed initially, while remaining litter residue starts to form soil organic matter (Melillo et al. 1989, Sjögersten and Wookey 2004). This prompts the question; how is carbon processed after this initial mass loss, bearing in mind that standing stocks of soil organic matter are very low in these forests (Hartley et al. 2012, Parker et al. 2015)? In boreal forests, ectomycorrhizal fungi (EMF) grow in the organic and mineral horizons below the litter (Lindahl et al. 2007) and have been shown to be able to stimulate decomposition of macromolecular complexes through the production of extracellular enzymes, specifically, peroxidases (Bödeker et al. 2014, Lindahl and Tunlid 2015). Although other pathways are plausible, we propose that the decomposition of litter in this forest ecosystem is characterized by an initial rapid mass loss due to metabolism by saprotrophic fungi and bacteria of relatively simple organic molecules, e.g., carbohydrates, and a subsequent steadier decomposition by EMF of the remaining more complex compounds. Taken together, this could result in a thin organic soil horizon and low net C storage in the ecosystem (Hartley et al. 2012).

This study has shown that litter of a common tundra heath species, *E. nigrum*, decomposes faster in forest or shrub environments than in tundra heath, and that this decomposition will be driven in the first instance by carbohydrate loss. As forests are expanding in range and cover in some areas of the sub‐Arctic (Tømmervik et al. 2009, Rundqvist et al. 2011, Hofgaard et al. 2013) and shrubs have been observed to be increasing in community dominance in many locations across the Arctic tundra (Tape et al. 2006, Myers‐Smith et al. 2011), the findings of the current study have important implications for the future of Arctic C stocks. If tundra heath soils, rich in less‐decomposed forms of C (Sjögersten et al. 2003), are colonized by deciduous forest, with its associated fungal community (including EMF, which are also potentially efficient decomposers [Lindahl and Tunlid 2015]), then this C will be rapidly metabolized and a significant part of the C currently stored in tundra heath will be released to the atmosphere. This would represent a positive feedback to climate warming.

In conclusion, the dominant litter types across the forest–heath ecotone decomposed faster in the most productive ecosystems. We hypothesize that this is due to a carbohydrate‐rich input of litter from the birch canopy and the presence of a decomposer community that can metabolize this relatively labile source of C. Using a snow fence experiment on tundra soils, we show that the effect of increased snow in the forest compared to the heath alone is modest and that the effect of environment on decomposition rates in the forest is likely exerted via microbial metabolism over the summer. We raise the hypothesis that microbially accessible litter C from tundra heath species is vulnerable to decomposition should more productive deciduous species further expand onto heaths, resulting potentially in a net emission of CO_2_ to the atmosphere.

## Data Availability

Data associated with this study are available from Figshare: https://doi.org/10.6084/m9.figshare.6724304.v1
