# Supplementary material for: Exploring drivers of litter decomposition in a greening Arctic: results from a transplant experiment across a treeline
Source: Ecology. 2018 Aug 15;99(10):2284–94. doi: 10.1002/ecy.2442 (PMC6849570; doi:10.1002/ecy.2442)
Supplement: Supplementary file 1 [file ECY-99-2284-s001.pdf]

Supporting Information. Exploring drivers of litter decomposition in a greening Arctic: Results from a transplant experiment across a tree-line. Thomas C. Parker, Jonathan Sanderman, Robert D. Holden, Gesche Blume-Werry, Sofie Sjögersten, David Large, Miguel Castro-Díaz, Lorna E. Street, Jens-Arne Subke, and Philip A. Wookey. *Ecology*. 2018.

Appendix S1.

Table S1. Partial Least-Squares Regression (PLSR) model statistics

Chemical shift region (ppm)	Dominant C form	Data range in calibration set	RMSE	R2
0 - 45	Alkyl	15.0-54.8	3.39	97.71
45 - 60	N-Alkyl/Methoxyl	4.2-8.6	0.91	48.11
60 - 95	O-Alkyl	17.8-49.0	3.36	94.57
95 - 110	Di-O-Alkyl	3.9-11.5	0.66	96.81
110 - 145	Aryl	6.6-12.9	1.49	43.94
145 - 165	O-Aryl	1.2-6.0	1.25	23.47
165 - 190	Amide/Carboxyl	1.0-13.7	2.04	54.62
190 - 215	Ketone	0.0-2.8	0.58	45.50

Table S2. Test statistics of three-way ANOVAs analysing the effect of species (*Betula pubescens* or *Empetrum nigrum* and site (Heath or Forest) on the mass remaining of different carbon components and the percentage of carbon components remaining compared to undecomposed samples.

	Mass of component			% mass remaining		
Component and factor	d.f	F	*P*	d.f	F	*P*
Carbohydrate						
Site	1,32	28.01	***< 0.001***	1,32	27.21	***< 0.001***
Species	1,32	26.35	***< 0.001***	1,32	7.66	***0.009***
Time	1,32	9.95	***0.003***	1,32	11.58	***0.002***
Site x Species	1,32	4.29	**0.047**	1,32	0.45	0.508
Site x Time	1,32	6.69	***0.014***	1,32	7.38	***0.011***
Species x Time	1,32	0.15	0.702	1,32	0.30	0.590
Site x Species x Time	1,32	0.44	0.510	1,32	0.00	0.982
Lipid						
Site	1,32	5.67	***< 0.001***	1,32	12.18	***0.002***
Species	1,32	0.41	***< 0.001***	1,32	18.36	***< 0.001***
Time	1,32	0.84	0.366	1,32	4.44	0.045
Site x Species	1,32	1.65	0.208	1,32	0.12	0.727
Site x Time	1,32	0.10	0.749	1,32	1.12	0.301
Species x Time	1,32	2.07	0.160	1,32	1.13	0.298
Site x Species x Time	1,32	0.38	0.544	1,32	1.78	0.194
Lignin						
Site	1,32	0.49	0.490	1,32	0.39	0.537
Species	1,32	0.00	0.986	1,32	1.49	0.230
Time	1,32	11.05	***0.002***	1,32	10.49	***0.003***
Site x Species	1,32	4.02	0.054	1,32	3.46	0.072
Site x Time	1,32	0.10	0.754	1,32	0.16	0.692
Species x Time	1,32	0.13	0.725	1,32	0.02	0.899
Site x Species x Time	1,32	2.81	0.103	1,32	2.91	0.098

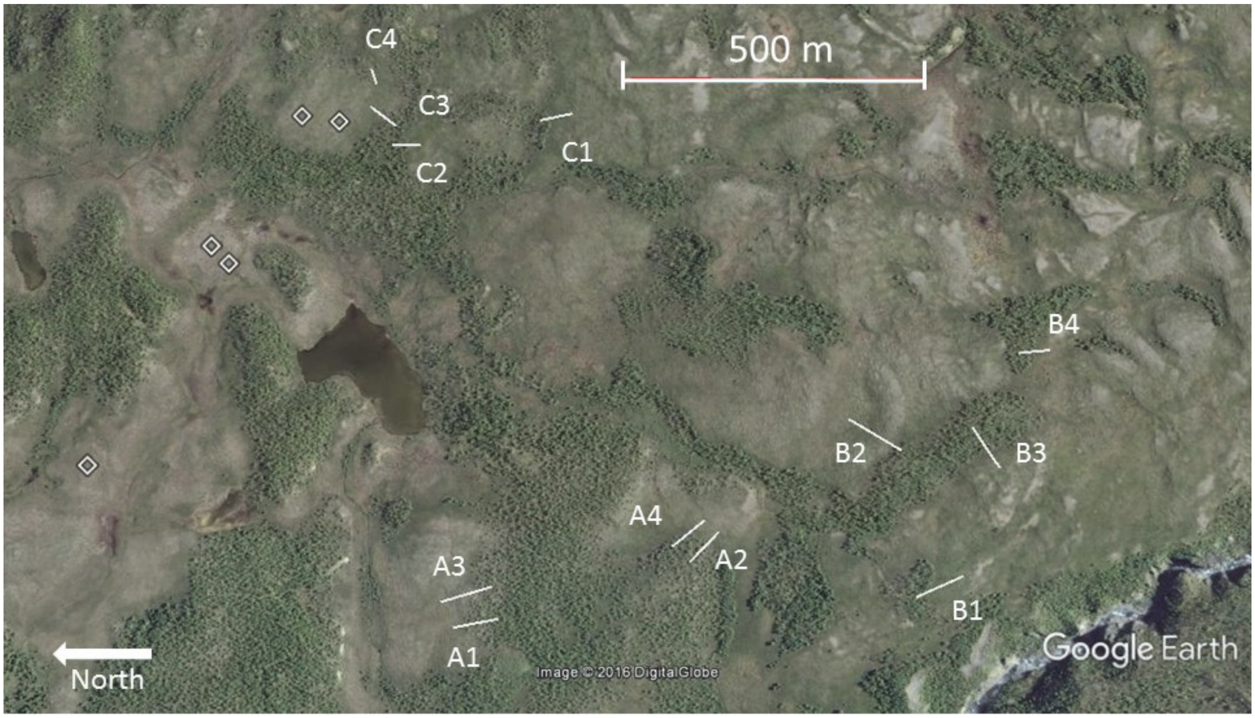

Figure S1. Google Earth image showing multiple transects across forest-heath ecotones at Abisko (Nissunsnuohkki). Labels represent the individual identifier for each transect. Snow fence locations are indicated by white diamonds to the north of the transects.

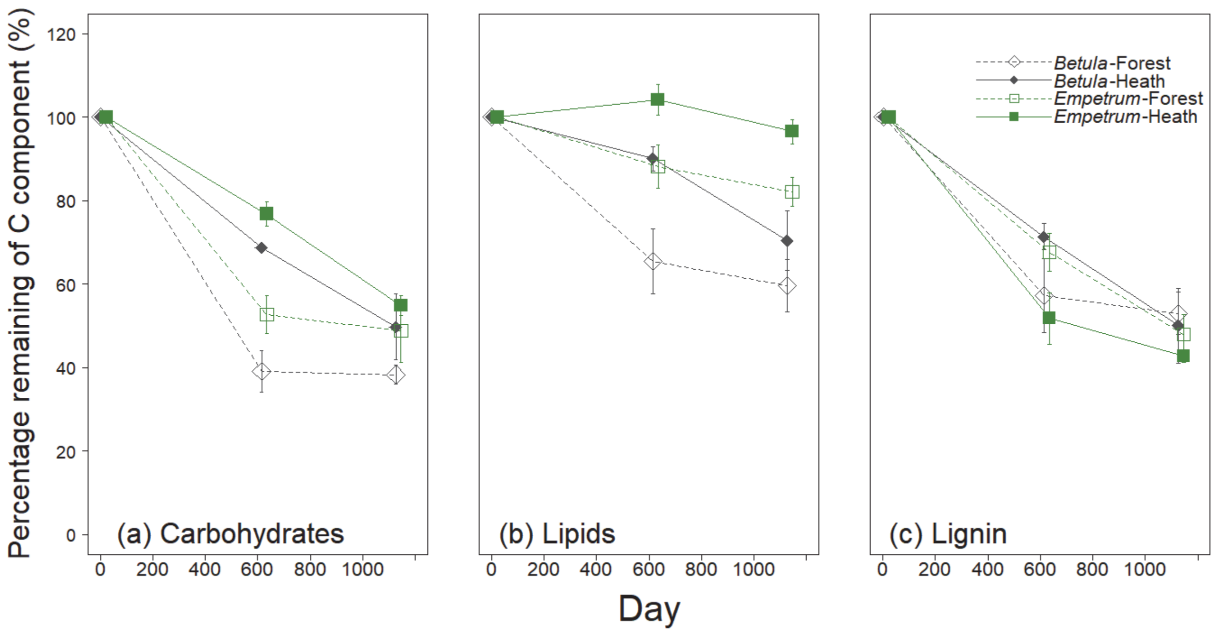

Figure S2. Percentage mass remaining compared to undecomposed litter of (a) Carbohydrates, (b) Lipids and (c) Lignin of *Betula pubescens* (grey diamonds) and *Empetrum nigrum* (black squares) in forest (open shapes) and heath (closed shapes) environments at initial levels (t0), after 614 days of decomposition (t4) and 1126 days of decomposition (t5). Error bars represent ± 1 SEM (controls: n = 3, decomposed samples: n = 5).
